# Nickel Toxicity Induced Changes in Nutrient Dynamics and Antioxidant Profiling in Two Maize (*Zea mays* L.) Hybrids

**DOI:** 10.3390/plants9010005

**Published:** 2019-12-18

**Authors:** Muhammad Amjad, Hasan Raza, Behzad Murtaza, Ghulam Abbas, Muhammad Imran, Muhammad Shahid, Muhammad Asif Naeem, Ali Zakir, Muhammad Mohsin Iqbal

**Affiliations:** Department of Environmental Sciences, COMSATS University Islamabad, Vehari-Campus, Vehari 61100, Pakistan; hasanbotanist@gmail.com (H.R.); behzadmurtaza@ciitvehari.edu.pk (B.M.); Ghulamabbas@ciitvehari.edu.pk (G.A.); imranrb@ciitvehari.edu.pk (M.I.); muhammadshahid@ciitvehari.edu.pk (M.S.); asif.naeem@cuivehari.edu.pk (M.A.N.); zakirali@cuivehari.edu.pk (A.Z.); mohsiniqbal6369@gmail.com (M.M.I.)

**Keywords:** maize hybrids, nickel, nutrients, translocation, oxidative stress

## Abstract

Nickel (Ni) is among the essential micronutrient heavy metals utilized by plants. However, an elevated level of Ni causes serious concerns for plants’ physiology and their survival. This study evaluated the mechanisms influencing the growth, physiology, and nutrient dynamics in two commercial maize hybrids (Syngenta and Pioneer) exposed to Ni treatments in hydroponics nutrient solution (NS). Seedlings were raised in plastic trays with quartz sand, and subsequently transferred to Hoagland’s NS at the two leaves stage. After three days of transplantation, Ni levels of 0, 20, and 40 mg L^−1^ were maintained in the nutrient solution. After 30 days of Ni treatments, seedlings were harvested and different growth, physiological, and nutrient concentrations were determined. The results showed that with increasing Ni concentration, the growth of maize hybrids was significantly reduced, and the maize hybrid, Pioneer, showed significantly higher growth than that of Syngenta at all levels of Ni. Higher growth in Pioneer is ascribed to elevated levels of antioxidant enzymes (SOD, CAT, GR, APX, and POX), lower damage to cellular membranes (i.e., higher MSI and lower MDA), and higher tissue nutrient concentrations (N, P, K, Ca, Mg, Fe, Mn, Zn, and Cu). Furthermore, the maize hybrids showed a difference in nutrient translocation from root to shoot which could be one of the factors responsible for differential response of these hybrids against Ni treatments.

## 1. Introduction

Heavy metal toxicity is among the major environmental issues reducing the yield of agricultural crops and posing serious health concerns for humans. Unlike other heavy metals, such as Cd, Pb, Hg, and Ag, Ni is an essential micronutrient that helps the urease enzyme convert urea into ammonia and carbon dioxide [1]. Ni deficiency can trigger inactivation of urease leading to urea accumulation to toxic levels which appear in the form of necrosis of leaf tips [2]. Nickel plays a very important role in plant physiology starting from germination to yield [3]. Nickel enhances yield and quality of most crops [4,5]. Some crops, such as barley (*Hordeum vulgare* L.), cannot complete their life cycle without Ni [6].

Excessive Ni, added to soil through irrigation with sewage sludge applied as a fertilizer or soil amendment, mining, and industrial processes, leads to its toxicity [7]. The worldwide average concentration of Ni in natural soils is 22 mg kg^−1^ [8,9] and has been reported up to 26.4 g kg^−1^ in soil and 0.3 mg L^−1^ in water [10,11]. Its toxicity symptoms appear between 0.19 and 0.85 mM kg^−1^ plant dry biomass [12]. Nickel toxicity inhibits the enzymatic activity, such as the Calvin cycle and chlorophyll biosynthesis which decreases photosynthetic efficiency in plants [13]. Moreover, Ni toxicity affects plants water status and the efficiency of antioxidative machinery [14,15].

Nickel toxicity shares toxic effects with other abiotic stresses such as the production of a large number of reactive oxygen species (ROS) [16]. The accumulation of reactive oxygen species in plants damages almost all the cellular components, for example, cell membrane, lipids, pigments, enzymes, chloroplasts, and nucleic acids [17,18]. The antioxidative enzymes such as superoxide dismutase (SOD), catalase (CAT), glutathione reductase (GR), and peroxidase (POX) etc., play their role in superoxide radical (O_2_^−^) and hydrogen peroxide (H_2_O_2_) and inhibit the formation of hydroxyl radicals (OH) [16,19]. Nickel, at an extremely low concentration (0.05 mM), enhances the activity of POD, SOD, and guaiacol peroxidase (GOPX) [20]. However, at elevated Ni concentrations (500 mg kg^−1^), antioxidative enzyme activity decreases and leads to oxidative damage in plants [21].

Nickel is reported to induce the deficiency of Fe and Zn and hinders the uptake of other heavy metals such as Cd, Co, Cr, and Pb [22]. Nickel toxicity lowers the concentration of N in the roots and leaves of mungbean and chickpea [23]. Hence, a severe toxicity has been observed whenever Ni was supplemented with other heavy metals such as Cd, Cu, Pb, and Zn [24]. The protein contents and carbohydrates decreased with Ni treatment (0, 200, 400, and 800 mg kg^−1^) in *Myplis snavelus* and sunflower [25], soyabean [26], and maize [27].

Nickel toxicity not only disrupts the uptake of important macro and micronutrients but also hinders the translocation of these nutrients from root to shoot, grain, and fruit [28]. The studies have reported that Ni toxicity tends to reduce the translocation of N from root to shoot [11,29]. Similarly, Ni interferes with translocation of micronutrients especially Fe being similar in chemical properties [30]. It has been reported that one of the causes of Fe deficiency under Ni toxicity is decrease in its translocation from root to shoot [22]. Moreover, Ni toxicity also negatively affects the assimilation of other nutrients [11]. 

Maize (*Zea mays* L.) is an important cereal crop which is cultivated all over the world. Maize hybrids have higher yield as compared with other varieties and genotypes. However, these hybrids need to be tested under different abiotic stresses such as salinity, drought, and metal toxicity etc. to evaluate their potential yield and survival for sustainability of the agriculture sector. Therefore, the current experiment was conducted to evaluate the physiological response, nutrient uptake, and translocation in two commercial maize hybrids (Syngenta and Pioneer) under Ni toxicity.

## 2. Materials and Methods

### 2.1. Selection of Plants and Growth Conditions 

The experimental site (latitude, 30°-1.9998′ N, longitude 72°-21′ E, and altitude 184.4 m) had average a minimum and maximum temperature of 15 to 26.4 °C and a relative humidity of 57.5% to 62.7%. Two hybrids of maize, “Pioneer-32F10” and “Syngenta-8441”, were selected for the experiment and seeds were purchased from the local market. These hybrids are commonly cultivated in Punjab by the farmers. 

Maize seedlings were raised in a 2 inch layer of acid washed quartz sand and irrigated with distilled water. The maize seedlings were transplanted to a Styrofoam sheet floating over Hoagland’s nutrient solution at the two leaves stage. The concentrations of different salts were: 5 mM KNO_3_, 5 mM Ca(NO_3_)_2_, 2 mM KH_2_PO_4_ and 1.5 mM MgSO_4_, 9.11 µM MnSO_4_, 1.53 µM ZnSO_4_, 0.235 µM CuSO_4_, 24.05 µM H_3_BO_3_, 0.1 µM Na_2_MoO_4_, and 268.6 µM Fe-EDTA. Aeration was maintained throughout the experimental period and pH was maintained daily between 6.0 and 6.5 by 1N NaOH/HCl solution. After three days of transplantation, Ni levels of 0, 20, and 40 mg L^−1^ were maintained in the NS using NiCl_2_.6H_2_O. The nutrient solution was changed after every week throughout the experimental period. 

### 2.2. Plant Biomass Measurements

The plants were sampled after 30 days of Ni treatments and plant growth parameters were measured. Shoot fresh weight of maize hybrids was measured with analytical balance, whereas root fresh weight was measured after paper blotting the roots to remove moisture. The shoot and root length was measured immediately, while their dry weight was measured after oven drying the samples at 65 °C for 48 h.

### 2.3. Determination of Antioxidant Enzymes Activity

Leaf sample (0.5 g) was homogenized with potassium phosphate buffer (pH 7.0) in a precooled mortar and pestle. The homogenate was centrifuged in a temperature-controlled centrifuge machine (4 °C) for 20 min at 10,000× *g*. The supernatant obtained was used for the determination of antioxidative enzymes. 

The activity of superoxide dismutase activity (SOD) was determined by measuring its ability to inhibit the photochemical reduction of nitroblue tetrazolium (NBT). The reaction mixture was prepared along with a control (without enzyme extract) as described by Gupta et al. [31]. The reaction was started by adding riboflavin and placing the mixture under light (15W) for 15 min. The absorbance was measured at 560 nm after stopping the reaction by switching off the light. The SOD activity was calculated in mg^−1^ protein min^−1^ (1 unit = 50% reduction in absorbance).

The catalase activity (CAT) was determined spectrophotometrically by recording the decrease in absorbance at 240 nm resulting from the decomposition of H_2_O_2_. The reaction mixture consisted of potassium phosphate buffer, H_2_O, and enzyme extract as explained by Aebi [32]. The enzyme activity was presented in mmol H_2_O_2_ mg^−1^ protein min^−1^. 

The activity of ascorbate peroxidase (APX) was measured by the oxidation of ascorbate and decrease in absorbance at 290 nm, according to the method described by Amako et al. [33]. The enzyme activity was expressed in mmol H_2_O_2_ mg^−1^ protein min^−1^.

Glutathione reductase activity (GR) was measured by the reduction of glutathione disulfide (GSSG) to two molecules of glutathione equivalent to NADPH coenzyme expressed as µmol of NADPH mg^−1^ protein min^−1^ by recording the increase in absorbance (412 nm for 5 min) according to Sairam et al. [34]. 

Peroxidase (POX) activity was determined by measuring its ability to cause reduction of guaiacol (recorded at 436 nm for 90 s) in the reaction mixture prepared according to the method described by Pandey and Pathak [14]. The enzyme’s activity was expressed as mmol H_2_O_2_ mg^−1^ protein min^−1^.

The Bradford method was used to measure the leaf protein contents [35].

### 2.4. Determination of Lipid Peroxidation 

Malondialdehyde contents (MDA) were measured to assess the extent of lipid peroxidation in maize leaves. Leaf sample (0.5 g) was homogenized in 5 mL of 0.1% trichloro acetic acid (TCA) and 4 mL of 0.5% thiobarbituric acid (TBA). The mixture was heated for 30 min at 95 °C and then, centrifuged at 20,000× *g* for 10 min. The supernatant was collected and the absorbance of reaction mixture was recorded at 532 and 600 nm [36]. The difference in absorbance was used to calculate the MDA content (nmol/g fresh weight).

### 2.5. Determination of Membrane Stability Index

The stability of leaf membrane was determined by measuring the electrical conductivities (EC) of two sets of double distilled water having 0.1 g of leaf sample at 40 °C after 30 min (EC_1_) and at 100 °C after 10 min (EC_2_) according to Amjad et al. [37]. The leaf membrane stability index (MSI) was calculated by the formula:

(1) $$MSI=\left(1-\frac{EC_{1}}{EC_{2}}\right)\times 100$$

### 2.6. Tissues Nitrogen and Phosphorus Determination

Total nitrogen concentration in shoot and root was analyzed with Kjeldahl digestion [38], whereas tissues total phosphorus concentration was measured using the colorimetric method [39].

### 2.7. Atomic Absorption Spectrometric Measurements

The concentrations of Ca, Mg, K, Fe, Zn, Cu, Mn, and Ni in shoot and root were determined on an atomic absorption spectrophotometer (Thermo AA, Solaar series, Thermo Scientific) [40]. The concentrations were determined from one-gram oven-dried samples after wet digestion with a di-acid mixture of HNO_3_ and HClO_4_ (1:3). 

Nickel uptake was calculated as:

Ni uptake (mg) = tissues Ni concentration × tissues dry mass(2)

While root to shoot (R-S) Ni translocation was calculated as:

R-S translocation = concentration of nutrient in shoot/concentration of nutrient in root(3)

### 2.8. Statistical Analysis

The experiment was performed according to factorial design replicated thrice. The significance of treatment means was determined by two-way analysis of variance (ANOVA). The least significant test was applied to check significant differences (LSD) among the treatment means using the computer program Statistix 8.1.

## 3. Results

### 3.1. Growth of Maize Hybrids

The results revealed that growth parameters (shoot/root fresh and dry weight and length) decreased significantly after thirty days of exposure to 20 and 40 mg Ni L^−1^ as compared with 0 mg L^−1^ Ni treatment in both maize hybrids (Table 1). Syngenta showed significantly higher values of measured growth parameters as compared with Pioneer under 20 and 40 mg L^−1^. However, the difference was not significant between the maize hybrids for shoot and root length at all levels of Ni and for shoot and root fresh weight at 0 mg L^−1^. 

### 3.2. Antioxidant Enzymes Activity

**Figure 1:**
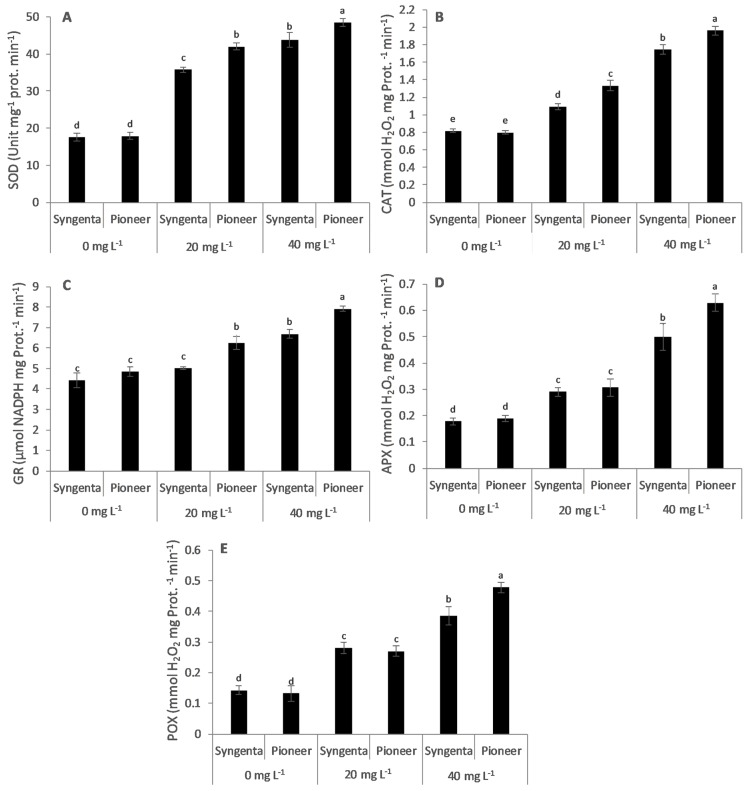
Effect of Ni treatments (0, 20, and 40 mg L^−1^) on activity of antioxidant enzymes: SOD (**A**), CAT (**B**), GR (**C**), APX (**D**), and POX (**E**) in two maize hybrids, Syngenta-8441 (Syngenta) and Pioneer-32F10 (Pioneer). Different letters on bars represent the result of LSD test, bars sharing the same letter(s) show the non-significance of the means at *p* < 0.05.

The maize hybrids showed a significant (*p* < 0.05) differential increase in antioxidant enzymes activity, i.e., SOD, CAT, GR, APX, and POX at 20 and 40 mg L^-1^ Ni as compared with the control (0 mg L^−1^ Ni) (Figure 1). At 20 mg L^−1^, the increase in activity of SOD, CAT, GR, APX, and POX was 2.4, 1.7, 1.3, 1.6, and 2 times, respectively, as compared with the control treatment in Pioneer, whereas in Syngenta the increase was 2.0, 1.3, 1.1, 1.6, and 2.0 times, respectively. Similarly, at 40 mg L^−1^, the increase in activity of SOD, CAT, GR, APX, and POX was 2.7, 2.4, 1.6, 3.3, and 3.6 times, respectively, as compared with the control treatment in Pioneer, whereas in Syngenta the increase was 2.5, 2.1, 1.5, 2.8, and 2.7 times, respectively.

### 3.3. Membrane Stability Index and Malondialdehyde Contents

**Figure 2:**
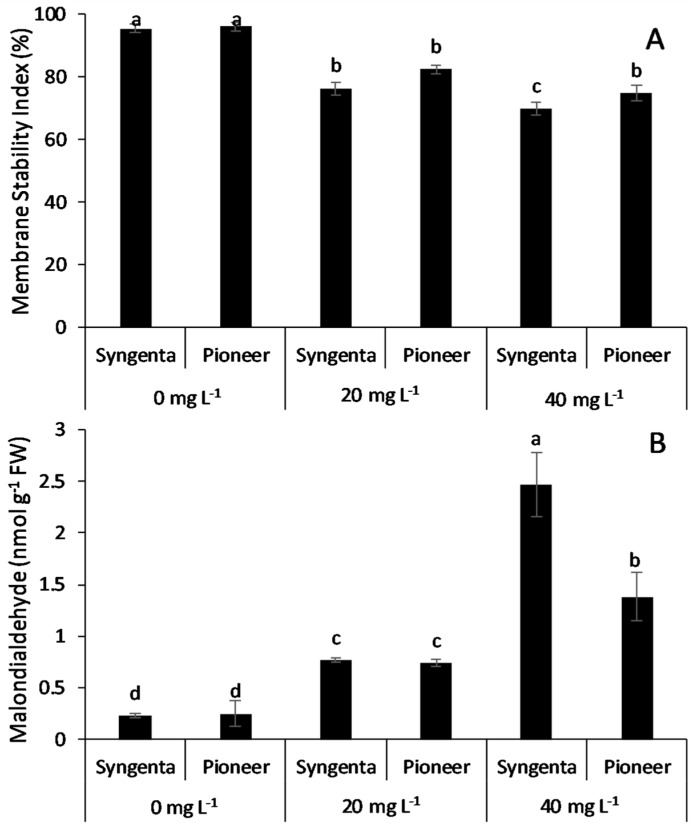
Membrane stability index (**A**) and lipid peroxidation (malondialdehyde) (**B**) in two maize hybrids, Syngenta (Syngenta-8441) and Pioneer (Pioneer-32F10), after 30 days of Ni treatment (0, 20, and 40 mg L^−1^). Different letters on bars represent the result of the LSD test, bars sharing the same letter(s) show the non-significance of the means at *p* < 0.05.

Membrane stability in both maize hybrids against Ni treatments (20 and 40 mg L^−1^) induced oxidative stress was measured by membrane stability index (%) and malondialdehyde concentration (Figure 2A,B, respectively). The damage caused by ROS to cellular membranes was assessed by measuring MDA contents produced as a result of lipid peroxidation. The results showed that MDA contents increased significantly with increasing Ni levels and correspondingly decreased the membrane stability index in both maize hybrids. The maize hybrids showed significant differences in terms of MDA concentration, relatively higher MDA content, and lower membrane stability index in Syngenta as compared with Pioneer. 

### 3.4. Nutrient Concentration in Tissues and Root to Shoot Translocation

**Figure 3:**
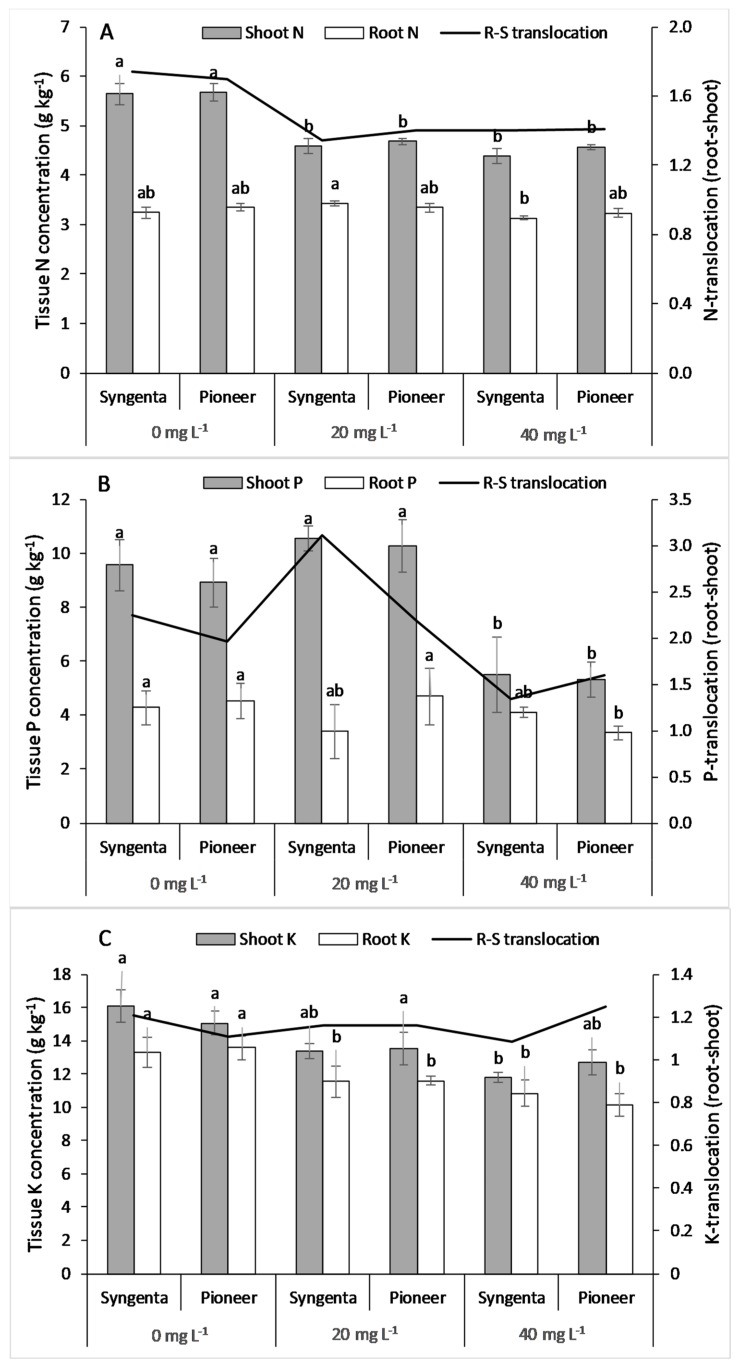
Concentrations of shoot and root N (**A**), P (**B**), K (**C**) and root to shoot translocation in two maize hybrids, Syngenta (Syngenta-8441) and Pioneer (Pioneer-32F10), after 30 days of Ni treatments (0, 20, and 40 mg L^−1^). Different letters on bars represent the result of the LSD test, bars sharing the same letter(s) show the non-significance of the means at *p* < 0.05.

The results showed a significant (*p* < 0.05) decline in shoot N concentration at 20 mg L^−1^ Ni concentration as compared with 0 mg L^−1^ Ni treatment. However, there was no significant difference in shoot N concentration between the 20 and 40 mg L^−1^ Ni level (Figure 3A), whereas root N concentration increased at the 20 mg L^−1^ and decreased at the 40 mg L^−1^ Ni level. Root to shoot N translocation decreased at the 20 mg L^−1^ Ni level and remained almost constant at the 40 mg L^−1^ Ni level. Maize hybrids (Syngenta and Pioneer) showed no significant difference for both shoot and root N concentration at all levels of Ni. 

The results showed no significant (*p* < 0.05) increase in P concentration at 20 mg L^−1^ Ni, whereas a significant decline in P concentration was observed at 40 mg L^−1^ Ni as compared with the control (Figure 3B). Root P concentration was not significantly affected by the Ni levels. The maize hybrids showed no significant differences at all Ni levels for both shoot and root P concentration. Root to shoot translocation of P increased at the 20 mg L^−1^ Ni level and decreased at the 40 mg L^−1^ level of Ni. 

Shoot and root K concentrations decreased significantly with increasing Ni toxicity (Figure 3C). The maize hybrids showed no significant differences for both shoot and root K concentrations at all levels of Ni. Root to shoot translocation of K was higher in maize hybrid Pioneer than that of Syngenta at 20 mg L^−1^ and 40 mg L^−1^ Ni. 

**Figure 4:**
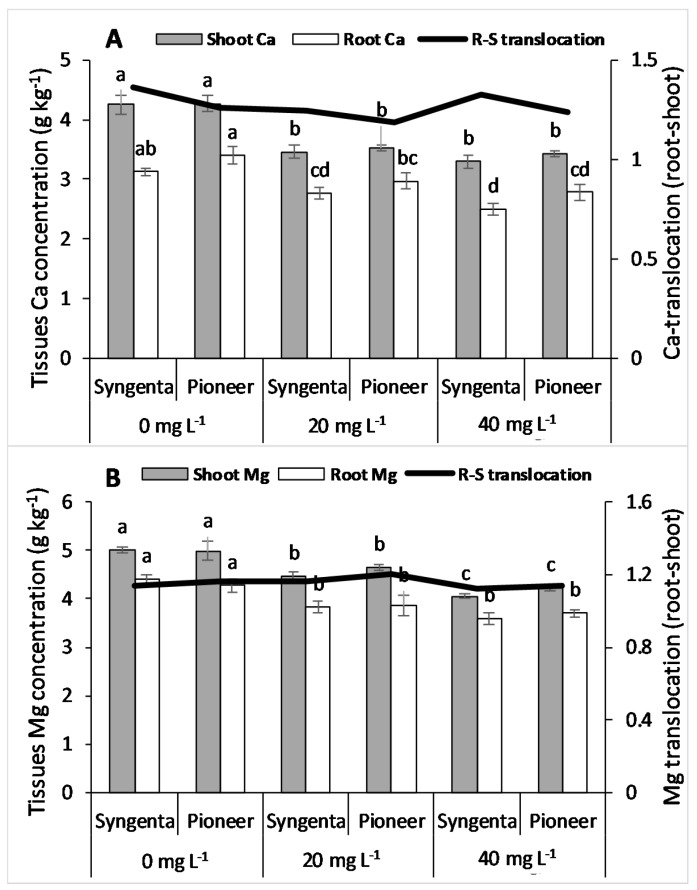
Concentration of shoot and root Ca (**A**), Mg (**B**), and root to shoot translocation in two maize hybrids, Syngenta (Syngenta-8441) and Pioneer (Pioneer-32F10), after 30 days of Ni treatments (0, 20, and 40 mg L^−1^). Different letters on bars represent the result of the LSD test, bars sharing the same letter(s) show the non-significance of the means at *p* < 0.05.

Both root and shoot tissues showed significantly low Ca concentration at elevated levels of Ni, i.e., 20 and 40 mg L^−1^ as compared with the control for both maize hybrids (Figure 4A). Root to shoot translocation of Ca was higher in Syngenta than that of Pioneer at all the Ni levels. Both of the maize hybrids showed a significant decreasing trend regarding shoot Mg concentration with increasing Ni level, whereas there was no significant difference between them at all the levels of Ni. In addition, root Mg concentration was significantly lower at the 20 and 40 mg L^−1^ Ni levels as compared with the control with no significant differences in the maize hybrids (Figure 4B), and Mg translocation (root to shoot) was higher in Pioneer than Syngenta at the 20 mg L^−1^ Ni level and decreased at the 40 mg L^−1^ Ni level. 

**Figure 5:**
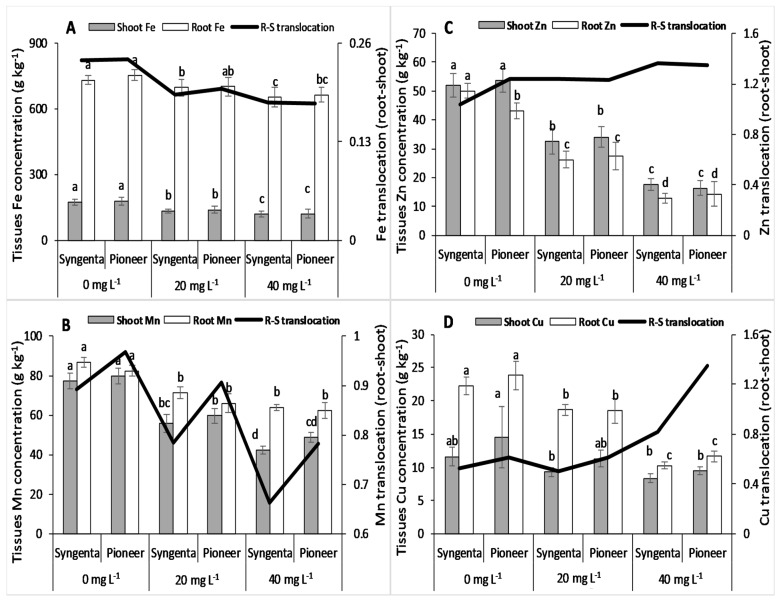
Concentration of shoot and root Fe (**A**), Mn (**B**), Zn (**C**), Cu (**D**), and their translocation (root to shoot) in two maize hybrids, Syngenta (Syngenta-8441) and Pioneer (Pioneer-32F10), after 30 days of Ni treatments (0, 20, and 40 mg L^−1^). Different letters on bars represent the result of the LSD test, bars sharing the same letter(s) show the non-significance of the means at *p* < 0.05.

Shoot Fe concentration decreased significantly, and there was no significant change in root Fe concentration in both maize hybrids with increasing Ni levels (Figure 5A). The maize hybrids showed no significant (*p* < 0.05) differences for both shoot and root Fe concentration at all the Ni levels. Root to shoot Fe translocation decreased at the 20 mg L^−1^ level of Ni as compared with the control.

The results showed that Mn concentration in tissues (shoot and root) decreased significantly with increasing Ni levels in both maize hybrids (Figure 5B). Translocation (root to shoot) of Mn decreased in both maize hybrids at the 20 mg L^−1^ Ni level with higher values in Pioneer than Syngenta. There was no significant difference in Zn concentration in tissues between maize hybrids at all the Ni levels (Figure 5C). Both shoot and root Zn concentration decreased significantly at 20 and 40 mg L^−1^ as compared with 0 mg L^−1^ Ni level, whereas its root to-shoot translocation increased. Both shoot and root Cu concentration decreased significantly (*p* < 0.05) by increasing Ni (20 and 40 mg L^−1^) levels in both maize hybrids (Figure 5D). The maize hybrids did not differ significantly in shoot and root Cu concentration at all Ni levels. Whereas, root to shoot translocation increased with increasing Ni levels.

### 3.5. Plant Tissues Nickel Concentration, Uptake, and Root to Shoot Translocation

**Figure 6:**
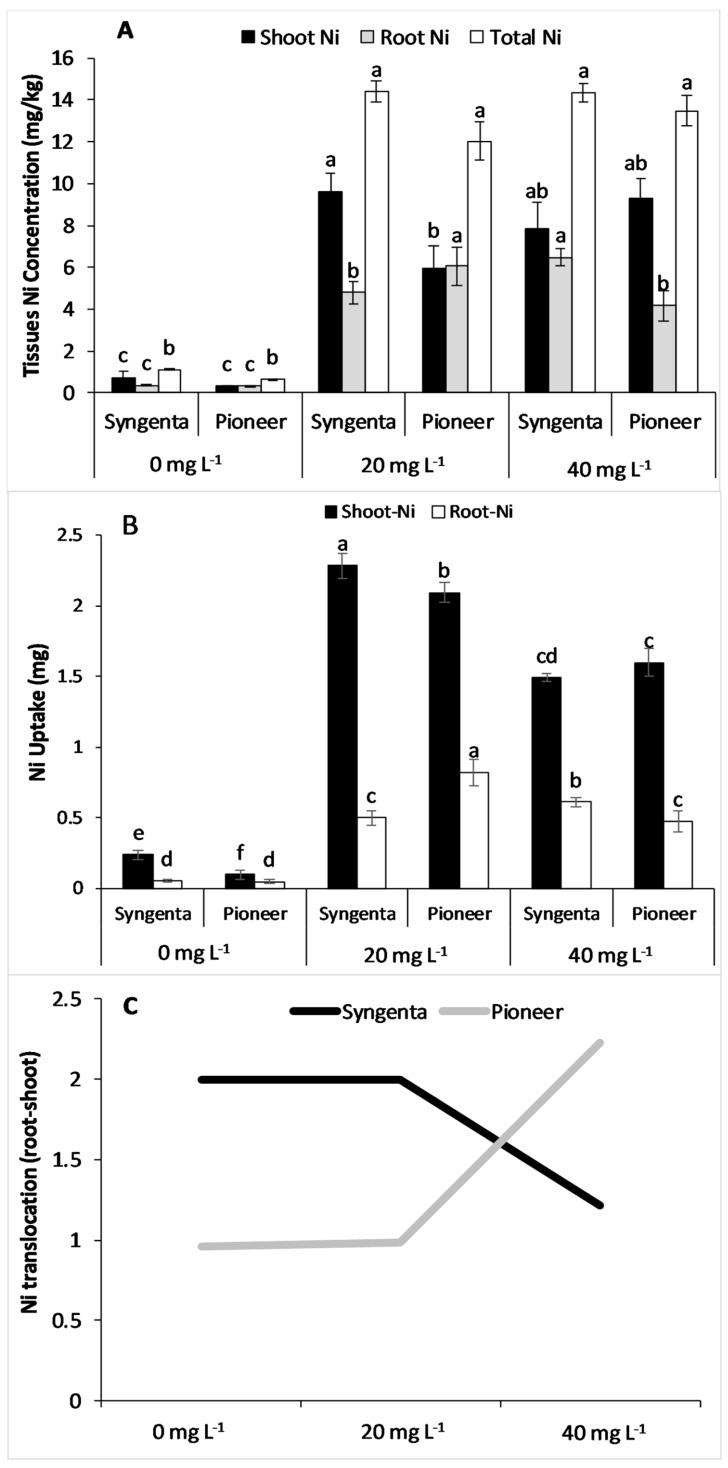
Concentration of shoot and root Ni concentration (**A**), uptake (**B**), and Ni root to shoot translocation (**C**) in two maize hybrids, Syngenta (Syngenta-8441) and Pioneer (Pioneer-32F10), after 30 days of Ni treatments (0, 20, and 40 mg L^−1^). Different letters on bars represent the result of the LSD test, bars sharing the same letters show the non-significance of the means at *p* < 0.05.

The results showed that total Ni concentration in the plants increased significantly with increasing Ni levels in both maize hybrids (Figure 6A). The maize hybrids differed significantly in terms of shoot and root Ni concentration especially at elevated Ni levels, i.e., 20 and 40 mg L^−1^. Shoot Ni concentration was significantly higher than root Ni in both maize hybrids except in Pioneer at 20 mg L^−1^. Shoot and root Ni uptake significantly increased at increasing Ni levels with significant differences between the maize hybrids (Figure 6B). Root to shoot Ni translocation remained almost constant at 0 and 20 mg L^−1^ in both maize hybrids although higher in Syngenta than Pioneer (Figure 6C) but decreased significantly in Syngenta and increased in Pioneer at the 40 mg L^−1^ Ni level. 

## 4. Discussion

This study focuses on the nutrient dynamics and antioxidant defense system of two maize hybrids, i.e., Syngenta and Pioneer against Ni toxicity in nutrient solution. The decrease in growth caused by Ni toxicity could be attributed to perturbed nutrient uptake and translocation because excess Ni decreases the uptake of macro and micronutrients [15,23]. Secondarily, the decrease in growth could also be attributed to oxidative damage caused by the excess of ROS measured by lipid peroxidation (malondialdehyde) and the membrane stability index. The results showed that the maize hybrid, Syngenta, had a higher concentration of malondialdehyde and a lower membrane stability index as compared with Pioneer, and thereby showed significantly higher growth in Pioneer than Syngenta at the 20 and 40 mg L^−1^ Ni levels (Table 1). Nickel toxicity has been reported to interfere with the plant water relations, wheat growth, and plant nutrient status [41]. Different plant species and genotypes show variation in survival ability against abiotic stress induced oxidative damage, i.e., malondialdehyde concentration is responsible for their tolerance against these stresses [42]. Similarly, in this experiment, maize hybrid Pioneer showed lower malondialdehyde concentration and higher membrane stability, thus, having higher growth than Syngenta (Figure 2). 

Nickel toxicity has been reported to cause damage to cellular membranes with the excessive production of ROS beyond the neutralizing capacity of the antioxidative system. Its toxicity is capable of oxidizing vital cellular membrane components including lipids, DNA, and proteins [43,44]. Nickel causes higher degrees of lipid peroxidation due to its higher mobility in plants as compared with other heavy metals’ toxicity (Co, Cd, Cu, and Zn). Similarly, this study showed that Ni (20 and 40 mg L^−1^ levels) caused the lipid peroxidation (MDA concentration), thereby decreasing MSI more in Syngenta as compared with Pioneer (Figure 2A,B). Lower lipid peroxidation in Pioneer could be attributed to the higher activity of SOD, CAT, GR, APX, and POX than in Syngenta. Nickel toxicity has been reported to enhance the activity of SOD which detoxifies superoxide ions to hydrogen peroxide and molecular oxygen [45,46]. Hydrogen peroxide is subsequently detoxified by other enzymes such as CAT, APX, and POX to H_2_O [47,48], whereas GR reduces oxidized glutathione (GSSG) to sustain the glutathione equilibrium in the cells [43]. The significantly higher activity of all the enzymes in the “Pioneer” maize hybrid as compared with the “Syngenta” indicated its higher Ni tolerance capability, responsible for better growth and lower lipid peroxidation (Table 1 and Figure 1). 

Owing to similar characteristics with both macronutrients (Ca, Mg) and micronutrients (Fe, Cu, and Zn), Ni competes with both of them in sorption and transpiration in plants [15,49]. Because of this similarity, a high concentration of Ni causes deficiency of these nutrients in plants by hindering their sorption, uptake, and translocation [30,41]. Our results validate previous findings, that Ni toxicity significantly decreased the nutrient concentrations in both shoot and root of maize hybrids. However, the Fe concentration in tissues remained almost constant with the excessive Ni in the growth medium (Figure 5A). Previous studies have reported a synergistic effect of Ni on N and P uptake and translocation from root to shoot in different plant species within the toxic range of Ni in the growth medium [2,50,51]. Contrarily, this study showed a decrease in root to shoot translocation of N, suggesting the toxicity of the applied Ni levels. The translocation of P showed an increase at the 20 mg L^−1^ level of Ni and a decrease at the 40 mg L^−1^ level of Ni, indicating strong competition between Ni and P ions at the 40 mg L^−1^ level of Ni. A significant difference in nutrient concentration between maize hybrids suggests that genotypes and hybrids vary in their ability to counter Ni toxicity. Moreover, a higher value of Ni translocation from root to shoot in “Pioneer” was one of the factors responsible for higher growth under Ni toxicity. Higher concentration and translocation of the measured micronutrients in the maize hybrid “Pioneer” could have enhanced the activity of SOD and CAT, since these metals are present in their prosthetic groups [28,44]. Lower values of these micronutrients (Fe, Mn, Cu, and Zn) in the maize hybrid Syngenta could have reduced the biosynthesis of the measured antioxidative enzymes, and therefore caused higher oxidative damage as compared with Pioneer.

Previous reports have suggested that Ni is a highly mobile element and approximately 50% of the plant-absorbed Ni is accumulated in roots, with over 80% in the vacuoles and 20% in cortex [11,23]. However, results from this study revealed that the Ni concentration was higher in the shoot than the root tissues and the total Ni concentration (shoot and root) did not remained at significantly high Ni levels in both maize hybrids (Figure 6A–C). These results suggest that total tissues Ni concentration does not remains significant beyond a certain level owing to the limited ability of maize hybrids to accumulate and uptake Ni from the growth medium. However, increased levels of Ni toxicity cause an opposite trend of root to shoot Ni translocation as suggested by the increase in root to-shoot translocation at the 40 mg L^−1^ level of Ni in tolerant maize hybrid Pioneer and sensitive (Syngenta). The increased Ni translocation at the 40 mg L^−1^ level of Ni in Pioneer is attributed to enhanced activity of the antioxidant enzymes resulting in lower lipid peroxidation and higher growth than that in Syngenta, as shown in Figure 1 and Figure 2. 

## 5. Conclusions

Results from this study suggest that Ni toxicity severely affects maize plant physiology with oxidative damage and disturbs the nutrient uptake and translocation in Pioneer and Syngenta maize hybrids. There are differences in maize hybrids regarding growth, antioxidant enzymes activity, nutrient uptake, and translocation. The higher growth in the maize hybrid “Pioneer” is attributed to elevated levels of antioxidative enzymes and nutrient translocation from root to shoot which affected the normal functioning of plants.

## Figures and Tables

**Table 1:** Effect of Ni treatments (0, 20, and 40 mg L^−1^) on shoot fresh weight, root fresh weight, shoot dry weight, root dry weight, shoot length, and root length of two maize hybrids, Syngenta-8441 (Syngenta) and Pioneer-32F10 (Pioneer). Each value is mean of three replicates + standard error. Values in the parentheses represent the % of the values in treatment (0 mg L^−1^) in both maize hybrids. The *p*-values show the significance for Ni levels, maize hybrids (MH), and their interaction (Ni x MH) at *p* < 0.05. Letters along the values show the results of the least significant test. Means that share the same letters show results that are not significant.

Ni Concentration	Hybrids	Shoot Fresh Weight (g) Mean ± S.E.	Root Fresh Weight (g) Mean ± S.E.	Shoot Dry Weight (g) Mean ± S.E.	Root Dry Weight (g) Mean ± S.E.	Shoot Length (cm) Mean ± S.E.	Root Length (cm) Mean ± S.E.
**0 mg L^−1^**	**Syngenta**	3.28 ± 0.26 a	2.26 ± 0.24 a	0.316 ± 0.019 b	0.135 ± 0.018 a	38.6 ± 0.95 a	31.6 ± 0.75 a
	**Pioneer**	2.64 ± 0.12 b	2.33 ± 0.11 a	0.388 ± 0.002 a	0.139 ± 0.012 a	37.6 ± 1.55 a	32.5 ± 3.30 a
**20 mg L^−1^**	**Syngenta**	2.16 ± 0.12 (66) c	2.13 ± 0.21 (94) ab	0.239 ± 0.016 (76) d	0.104 ± 0.009 (77) b	33.1 ± 1.64 (86) b	27.7 ± 1.44 (88) b
	**Pioneer**	2.50 ± 0.05 (95) bc	2.00 ± 0.13 (86) abc	0.351 ± 0.013 (90) c	0.131 ± 0.005 (94) ab	35.7 ± 1.61 (96) ab	28.8 ± 1.80 (89) b
**40 mg L^−1^**	**Syngenta**	1.31 ± 0.06 (40) e	1.50 ± 0.05 (66) bc	0.190 ± 0.005 (60) f	0.094 ± 0.007 (70) c	30.4 ± 1.78 (79) c	24.2 ± 0.92 (77) c
	**Pioneer**	1.72 ± 0.02 (65) d	1.65 ± 0.08 (71) c	0.272 ± 0.017 (70) e	0.111 ± 0.010 (80) abc	31.1 ± 3.40 (83) bc	26.0 ± 3.07 (80) bc
***p*-value**	**Ni**	0.0000	0.0018	0.0000	0.0132	0.0012	0.2104
	**MH**	0.0253	0.0357	0.0263	0.0345	0.0282	0.3312
	**Ni x MH**	0.0047	0.0772	0.0001	0.0425	0.1532	0.2461
